# Supplementary material for: Parent and Caregiver Perspectives towards Cannabidiol as a Treatment for Fragile X Syndrome
Source: Genes (Basel). 2022 Sep 6;13(9):1594. doi: 10.3390/genes13091594 (PMC9498854; doi:10.3390/genes13091594)
Supplement: Supplementary file 1 [file genes-13-01594-s001.zip › genes-1872872-supplementary.pdf]

Fragile X Syndrome Parent/Caregiver Survey

Screening questions:

1. Has the person been diagnosed with Fragile X syndrome (FXS)?
   - □ Yes
   - □ No – thank you for your time, to be eligible to participate the person must be diagnosed with FXS

2. Is the person currently taking a cannabidiol (CBD) supplement?
   - □ Yes (– the respondent goes on to complete the full questionnaire beginning with the "Demographic Questions" section)
   - □ No (– the respondent only answers the following question (a), Demographic Questions, and Medical Information):

   a. Have you considered giving the person a CBD supplement?
      - □ No – Thank you for your time, to be eligible to participate CBD supplements must have been taken or considered
      - □ Yes
        - o Considered but not given:
          - ▪ Please select any reasons why you have chosen not to give CBD supplements: (select all that apply)
            - The CBD is too expensive
            - I do not know where to get CBD
            - I did not think CBD would help with FXS symptoms
            - I do not know enough about CBD, so I was not comfortable giving it to the person
            - A doctor advised not to give the person CBD
            - Other people said not to give the person CBD
            - Other, please explain: _______________
        - o Given in the past and stopped:
          - ▪ Please select any reasons why you stopped giving CBD supplements: (select all that apply)
            - The CBD is too expensive
            - I did not know where to continue getting CBD
            - The CBD did not help with the FXS symptoms
            - I do not know enough about CBD, so I did not feel comfortable continuing to give it to the person
            - The person experienced side effects while taking the CBD
            - A doctor advised to stop giving the person CBD
            - Other people said to stop giving the person CBD
            - Other, please explain: _______________

Demographics Questions

1. What is the age of the person with FXS? ______________

2. What is the sex of the person?
- □ Female
- □ Male
- □ Other, please specify: ___________

3. Which ethnic background(s) best describe him/her? (select all that apply)
   1. White
   2. Black or African-American
   3. Hispanic or Latino
   4. American Indian or Alaskan Native
   5. Asian
   6. Native Hawaiian or other Pacific Islander
   7. I would prefer not to answer
   8. Other ethnic background (please describe): ______________________________________

4. What is the annual household income of the primary guardian(s)? (If applicable, combine household incomes) (check one)
- □ < $25,000
- □ $25,000 to $49,999
- □ $50,000 to $74,999
- □ $75,000 to $99,999
- □ $100,000 to $149,999
- □ $150,000 or more
- □ I would prefer not to answer

Medical Information

1. Does the person have any of the following? If so, please indicate the severity:

Matrix: None, Very mild, Mild, Moderate, Severe
- □ Intellectual Developmental Disability (IDD)
- □ Anxiety
- □ Seizures
- □ Hypersensitivity/overreaction to stimuli
- □ Hyperactivity
- □ Attention problems
- □ Autism Spectrum Disorder
- □ Irritability
- □ Aggression
- □ Self-injury
- □ Sleep problems/disorders (insomnia, sleep apnea)

2. Is the person currently taking any of the following prescription medications?
Matrix: Currently taking this medication, Took this medication in the past
- ☐ Stimulants (i.e. methylphenidate [Ritalin, Concerta, Methylyn, Metadate, Focalin, Aptensio, Daytrana, Quillivant], amphetamines and mixed amphetamine salts [Adderall], lisdexamphetamine [Vyvanse], dextroamphetamine [Dexedrine], atomoxetine [Strattera], modafinil [Provigil])
- ☐ SSRIs (i.e. fluoxetine [Prozac], sertraline [Zoloft], paroxetine [Paxil], citalopram [Celexa], escitalopram [Lexapro])
- ☐ Non-SSRI antidepressants (i.e. trazodone, bupropion [Wellbutrin], venlafaxine [Effexor], tricyclics [amitriptyline, imipramine, etc])
- ☐ Alpha-agonists (i.e. clonidine [Catapres, Kapvay], guanfacine [Tenex, Intuniv])
- ☐ Antipsychotics (i.e. aripiprazole [Abilify], risperidone [Risperdal], haloperidol [Haldol], quetiapine [Seroquel], ziprasidone [Geodon], olanzapine [Zyprexa], thioridazine [Mellaril])
- ☐ Mood stabilizers (i.e. Lithium, valproic acid [Depakote], topiramate [Topamax], carbamazepine [Tegretol], oxcarbazepine [Trileptal], lamotrigine [Lamictal])
- ☐ Anxiolytics (i.e. clonazepam [Klonopin], diazepam [Valium], lorazepam [Ativan], alprazolam [Xanax], buspirone [Buspar, Vanspar], benzodiazepines)
- ☐ Acamprosate
- ☐ Minocycline
- ☐ Metformin
- ☐ Lovastatin

3. Is the person currently taking any other medications (not included above) or any non-CBD supplements? Please list: __________________

Form, Type, Brand, Dose, and Frequency of CBD Use

1. What form of CBD supplement is used? (check all that apply)
- ☐ CBD oil or tincture
- ☐ CBD butter
- ☐ CBD gummies or edibles (lollipops, brownies, soda)
- ☐ CBD capsules or pills
- ☐ CBD smoke or vapor
- ☐ Topical CBD lotion/cream
- ☐ CBD Zygel (Zynerba clinical trial)
- ☐ Epidiolex (CBD prescribed to treat some forms of epilepsy)
- ☐ Other, please describe: __________________

2. What type of CBD supplement is used? (check all that apply)
- ☐ Pure CBD: The supplement only contains CBD
- ☐ Broad spectrum: In addition to CBD the supplement also contains the other compounds that are naturally occurring in the cannabis plant, except traces of THC have been removed
- ☐ Full spectrum: In addition to CBD the supplement contains all the other compounds that are naturally occurring in the cannabis plant, including traces of THC (<0.3%)
- ☐ CBD Gel Compound (Zygel, Zynerba clinical trial)
- ☐ Epidiolex CBD Compound
- ☐ I do not know
- ☐ Other, please describe: __________________
- ☐ I choose not to answer this question

3. What is the brand of CBD currently used?
   ____
4. If possible, please upload a photo of the CBD supplement bottle/label:

5. How did you decide on this brand?
   ____
6. What is the concentration of the CBD product used, if known (mg. per bottle, mg. per mL, mg. per gummy, etc.):
   ____

7. About how many mg. of CBD are given at a time?
   ____

8. How often does he/she take CBD, on average?

☐ Everyday
☐ Almost every day
☐ 2-4 times a week
☐ Once a week
☐ Less than once a week

9. How many times per day does he/she take CBD, on average?

☐ Once per day
☐ Twice per day
☐ Three times per day
☐ More than three times per day
☐ Other, please describe: __________________

10. How long has he/she been taking CBD?

☐ Less than 1 month
☐ 1 – 6 months
☐ 7– 12 months
☐ 1 – 2 years
☐ 3 – 5 years
☐ More than 5 years

11. Have you tried other brands of CBD in the past? Please list any brands previously used, and explain why you stopped using them:
    ____

Rationale for Use: The next questions ask about why the person with FXS is taking CBD

1. Did he/she start using CBD to treat the following? (check all that apply)

☐ Intellectual Developmental Disability (IDD)
☐ Anxiety
☐ Seizures
☐ Hypersensitivity/overreaction to stimuli
☐ Hyperactivity
☐ Attention problems
☐ Autism Spectrum Disorder

- ☐ Irritability
- ☐ Aggression
- ☐ Self-injury
- ☐ Sleep problems/disorders (insomnia, sleep apnea)
- ☐ Other, please describe: ___________________

2. Please indicate how much you agree with the following reasons for giving CBD supplements (if applicable):
   Matrix: Strongly Disagree, Disagree, Neutral, Agree, Strongly Agree

- ☐ I feel CBD is a more natural option than prescribed drugs
- ☐ I heard positive things about CBD supplements from a friend or from someone in my community
- ☐ I heard positive things about CBD supplements from a medical provider
- ☐ Other treatments/ medications have not been very effective
- ☐ CBD has fewer side effects than other medications
- ☐ CBD supplements have provided added benefits
- ☐ I was able to obtain and try CBD supplements without a prescription
- ☐ I do not trust pharmaceutical companies
- ☐ I do not trust FDA approved medications

3. Are there any other reasons (not included above)? Please explain:
____

Perception of Effects: The next questions ask about the effects seen while the person is taking CBD supplements

1. What changes have you seen during CBD use in each of the following?

(Check "not applicable" if the person did not experience that symptom/condition prior to CBD use):
Matrix: Very Much Improved, Much Improved, Minimally Improved, No Change, Minimally Worse, Much Worse, Very Much Worse, Not Applicable

- ☐ Intellectual Developmental Disability (IDD)
- ☐ Anxiety
- ☐ Seizures
- ☐ Hypersensitivity/
- ☐ overreaction to stimuli
- ☐ Hyperactivity
- ☐ Attention problems
- ☐ Autism Spectrum Disorder
- ☐ Irritability
- ☐ Aggression
- ☐ Self-injury
- ☐ Sleep problems/disorders (insomnia, sleep apnea)

2. What changes have you seen during CBD use in each of the following behaviors?

(Check "not applicable" if the person did not show that behavior prior to CBD use):
Matrix: Very Much Improved, Much Improved, Minimally Improved, No Change, Minimally Worse, Much Worse, Very Much Worse, Not Applicable

- ☐ Screaming or yelling inappropriately
- ☐ Temper tantrums/outbursts

☐ Irritability and whining
☐ Depressed mood
☐ Needing their demands met immediately
☐ Crying over minor annoyances and hurts
☐ Mood changing quickly
☐ Stamping feet, banging objects, or slamming doors
☐ Seeking isolation from others
☐ Withdrawn and preferring solitary activities

3. Please comment further on any other effects/changes (not included above) that have been seen during CBD use: ___________________________

4. Would the person stop using CBD to participate in a FXS clinical trial?
☐ Yes
☐ No
☐ I do not know
☐ I would prefer not to answer

5. How would you feel if the person had to stop using CBD?
☐ Not at all disappointed
☐ Slightly disappointed
☐ Moderately disappointed
☐ Very disappointed
☐ Extremely disappointed

6. Overall, how much have the CBD supplements made a difference for the person's symptoms?
☐ Very much improved
☐ Much improved
☐ Minimally improved
☐ Neutral
☐ Minimally worse
☐ Much worse
☐ Very much worse
☐ I do not know

Side Effects

1. Please indicate if the person has experienced any of the following side effects, and at which severity, while taking CBD:
   Matrix: None, Very mild, Mild, Moderate, Severe

☐ Restlessness
☐ Irritability
☐ Sleepiness
☐ Sleep problems
☐ Paranoia (unrealistic distrust of others)
☐ Psychosis (disruptions to a person's thoughts and/or perceptions that make it difficult for him/her to recognize what is real and what is not)
☐ Change in appetite and/or weight

2. Are there any other side effects he/she has experienced while taking CBD? Please explain:

____

Financial

1. How much do you spend on CBD per month?

☐ Less than $50
☐ Between $50 and $100
☐ Between $100 and $200
☐ Between $200 and $300
☐ Between $300 and $400
☐ Between $400 and $500
☐ More than $500
☐ Prefer not to say

2. How do you feel about the cost of CBD supplements?

☐ The cost is reasonable
☐ Neutral
☐ The cost is unreasonable
☐ I choose not to answer this question
☐ Other, please explain: _________

Final Open-Ended Question

Is there anything else you can tell us about your experiences using CBD to treat FXS symptoms?
